# Stepwise Evolution and Exceptional Conservation of ORF1a/b Overlap in Coronaviruses

**DOI:** 10.1093/molbev/msab265

**Published:** 2021-09-10

**Authors:** Han Mei, Sergei Kosakovsky Pond, Anton Nekrutenko

**Affiliations:** 1 Department of Biochemistry and Molecular Biology, The Pennsylvania State University, University Park, PA, USA; 2 Department of Biology, Institute for Genomics and Evolutionary Medicine, Temple University, Philadelphia, PA, USA

**Keywords:** SARS-COV-2, frameshift, conservation

## Abstract

The programmed frameshift element (PFE) rerouting translation from *ORF1a* to *ORF1b* is essential for the propagation of coronaviruses. The combination of genomic features that make up PFE—the overlap between the two reading frames, a slippery sequence, as well as an ensemble of complex secondary structure elements—places severe constraints on this region as most possible nucleotide substitution may disrupt one or more of these elements. The vast amount of SARS-CoV-2 sequencing data generated within the past year provides an opportunity to assess the evolutionary dynamics of PFE in great detail. Here, we performed a comparative analysis of all available coronaviral genomic data available to date. We show that the overlap between *ORF1a and ORF1b* evolved as a set of discrete 7, 16, 22, 25, and 31 nucleotide stretches with a well-defined phylogenetic specificity. We further examined sequencing data from over 1,500,000 complete genomes and 55,000 raw read data sets to demonstrate exceptional conservation and detect signatures of selection within the PFE region.

Coronaviruses have large 26–32 kbp positive-strand RNA genomes. The initial ⅔ of the genome is occupied by an open reading frame (ORF) *ORF1ab* encoding nonstructural proteins essential for the coronaviral life cycle. As the designation “ab” suggests, it contains two reading frames with the 3′-end of *ORF1a* overlapping with the 5′-terminus of *ORF1b*. *ORF1b* is in −1 phase relative to ORF1a and translated via the −1 programmed ribosomal frameshifting controlled by the PFE. As *ORF1b* encodes crucial components of coronavirus transcription/replication machinery, including the RNA-dependent RNA polymerase (RdRp), disrupting PFE abolishes viral replication completely (Brierley 1995; Plant et al. 2010; Sola et al. 2015; Kelly et al. 2020). PFE consists of three consecutive elements: 1) an attenuator loop, 2) the “NNN WWW H” slippery heptamer, and 3) a pseudoknot structure (Kelly et al. 2020; Huston et al. 2021). The sequence and structural conformation of these elements determine the efficiency of the frameshift event, which ranges from 15% to 30% in SARS-CoV and SARS-CoV-2 (Baranov et al. 2005; Kelly et al. 2020). Because disruption of PFE arrests viral replication, it is a promising therapeutic target. As a result, a number of recent studies have scrutinized its characteristics (reviewed in Rangan et al. (2021)) revealing a fluid secondary structure (Iserman et al. 2020; Ziv et al. 2020; Huston et al. 2021). In addition to secondary structures, PFE harbors the overlap between *ORF1a and ORF1b*. It is defined as the stretch of sequence from “H” in the slippery heptamer to the stop codon of *ORF1a*. The position of the *ORF1a* stop codon determines overlap length. For example, in SARS-CoV-2, it is 16 bp, while in mouse hepatitis virus (MHV) it is 22 nt (Plant et al. 2010).

Our group has been interested in the evolutionary dynamics of overlapping coding regions (Nekrutenko et al. 2005; Chung et al. 2007; Szklarczyk et al. 2007). The vast amount of newly generated sequence and functional data—a result of the current SARS-CoV-2/COVID-19 pandemic—provides an opportunity to re-examine our current knowledge. The length of the *ORF1a and ORF1b* overlap is phylogenetically conserved. It evolved in a stepwise manner, where the changes in the overlap length are results of the loss of *ORF1a* stop codons leading to *ORF1a* extension, and the acquisition of insertions and deletions causing early stops of ORF1a.

Distance-based methods had shown that the δ-coronavirus genus was an early split-off lineage compared to α-, β-, and γ-coronavirus (fig. 1). Comparisons of the RdRp, 3CL^pro^, HEL, M, and N proteins suggested that γ- was more closely related to δ-coronavirus, while α- and β-coronavirus cluster together forming a distant clade (de Groot et al. 2012; Lau et al. 2012; Woo et al. 2012; Coronaviridae Study Group of the International Committee on Taxonomy of Viruses 2020). However, comparing the S protein trees, α- and δ-coronavirus share a higher amino acid identity, while β- and γ-coronavirus cluster together (Lau et al. 2012). Due to this, we initially assumed that α, β, and γ formed an unresolved trifurcation (fig. 1). To assess all possible configurations within this region, we surveyed all genomic sequences of family Coronaviridae available from the National Center for Biotechnology Information (NCBI; see Materials and Methods section). The distribution of overlap lengths among 4,904 coronaviral genomes (supplementary table S1, Supplementary Material online) is shown in supplementary fig. 1, Supplementary Material online. There are five distinct overlap length groups (7, 16, 22, 25, and 31 nt) with clear taxonomic specificity.

**Fig. 1.**
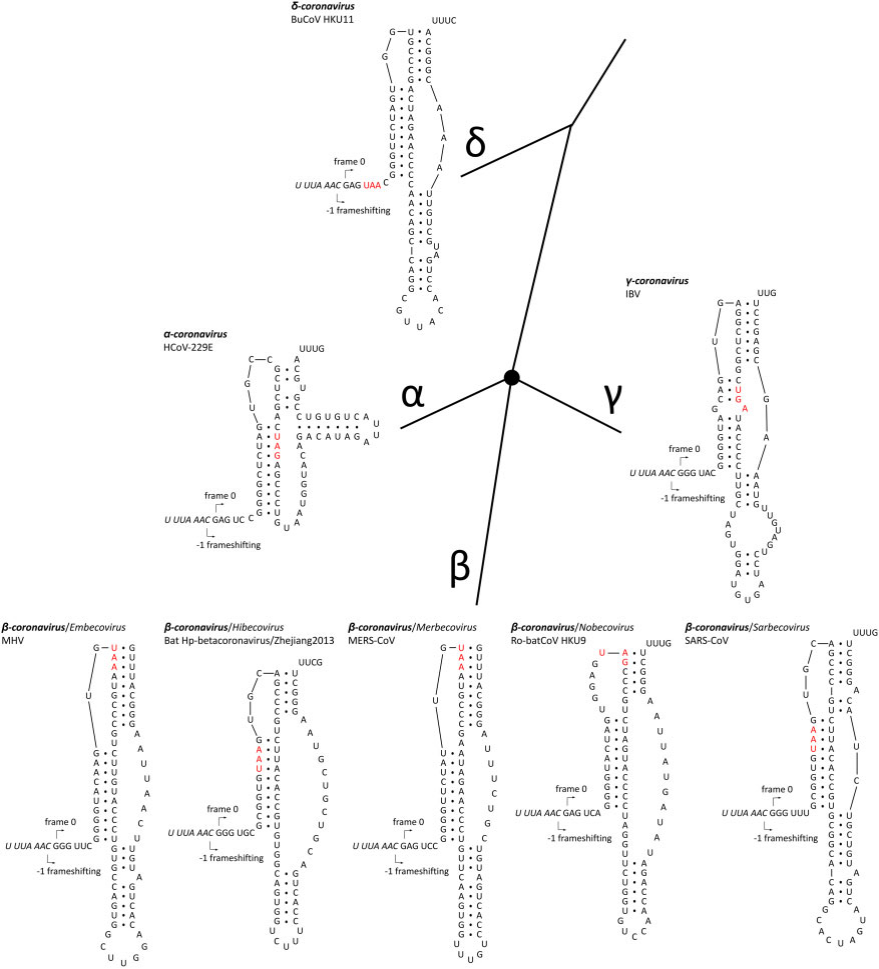
PFE slippery sites and pseudoknot structures in coronaviruses. The slippery site “UUUAAAC” is shown in italic. The *ORF1a* stop codon is shown in red. *ORF1b* frame starts from the “C” in the slippery site. *α-, β-*, and *γ-coronavirus* were plotted as splitting from one common node (black filled circle), with no phylogenetic order shown. The pseudoknot structures of SARS-CoV and MHV are redrawn based on Plant et al. (2010). The pseudoknot structures of HCoV-229E and IBV are redrawn based on Plant et al. (2005). HCoV-229E, human coronavirus 229E; NC_002645.1. MHV, mouse hepatitis virus; NC_001846.1. Bat Hp-β-coronavirus/Zhejiang2013, NC_025217.1. MERS-CoV, NC_019843.3. Ro-batCoV HKU9, rousettus bat coronavirus HKU9, NC_009021.1. SARS-CoV, NC_004718.3. IBV, infectious bronchitis virus, NC_001451.1. BuCoV HKU11, bulbul coronavirus HKU11, NC_011547.1.

We then compared the first 15 amino acids of *ORF1b* in all 4,904 entries (fig. 2). The amino acid sequences are highly conserved: positions 1 (R), 2 (V), 4 (G), 7 (S), 11–13 (ARL), and 15 (P) are almost invariable and highly redundant. Next, we compared the underlying nucleotide sequences of the PFE region (fig. 3). This suggests the following potential series of evolutionary events. δ-Coronavirus with 7 nt overlap most likely represents the ancestral state. Comparing coronaviruses with 7 nt (δ-coronavirus) and 31 nt (α- and γ-coronavirus) in the overlap, the stop codon which defines a 7 nt overlap is abolished at positions 5–7, through substitutions, which extends ORF1a to the next available stop codon at positions 38–40. This extension results in a new overlap with 31 nt in length (fig. 3*A*). Comparing coronaviruses with 31 nt (α- and γ-coronavirus) and 25 nt (β-coronavirus/Nobecovirus) overlaps reveals a “GTA” insertion at positions 28–30. “TA” from the “GTA” together with the following “G” forms a new stop codon leading to a 31 → 25 nt shortening of the overlap. In a Nobecovirus with a 25 nt overlap, the 31 nt overlap stop codon (at positions 38–40) is still observable (fig. 3*B*). Further comparison of coronaviruses with 31 nt (α- and γ-coronavirus) and 22 nt (β-coronavirus/Embecovirus and Merbecovirus) overlaps revealed a “GTA” insertion as well, but at positions 22–24. “TA” at positions 23–24 and the following “A” or “G” at position 25 constitute a new stop codon. In the 22 nt overlap, substitutions have been observed at the original stop codon (at positions 38–40) from 31 nt overlap coronaviruses; more specifically, “C” appears at position 39 (fig. 3*C*). Finally, we compared coronaviruses with 31 and 16 nt length in the overlap. The same “GTA” insertion footprint was found, at positions 16–18 ahead of the two “GTA” insertions in 31 → 25 nt and 31 → 22 nt events. “TA” at positions 17–18 and the following “A” at position 19 form the stop codon in the 16 nt overlap coronaviruses. In addition, deletions at positions 13–15 were observed (fig. 3*D*). These deletions are referred as “TCT”-like, since “TCT” are the dominant nucleotides observed at positions 13–15 in the 7 and 31 nt overlap coronaviruses. At positions 38–40, the ancestral stop codon in the 31 nt overlap coronaviruses cannot be seen, since the nucleotide at position 39 is invariably represented by “T” (fig. 3*D*). The variable position of the stop codon likely has an implication to the frameshift efficiency in these taxa as was shown by Bhatt et al. (2021). These authors demonstrated that extension of the distance between the slippery heptamer and the stop codon of 0-frame decreases frameshifting frequency: an increase in the distance by 15 nucleotides, as is the case in α- and γ-coronaviruses (fig. 3), decreases efficiency by ∼20%, while removal of the stop decreases it by half.

**Fig. 2.**
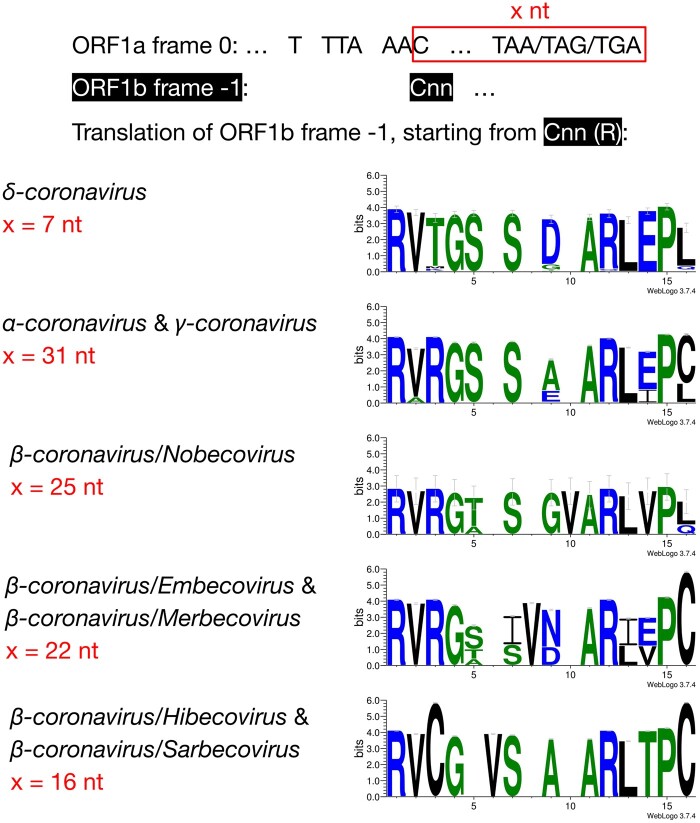
Amino acid alignment of the first 13–14 amino acids in coronaviruses with different lengths in the overlap region. For each genus/subgenus shown, all coronavirus entries belonging to it were used to generate the consensus amino acid sequences. Gaps are included to maintain alignment.

**Fig. 3.**
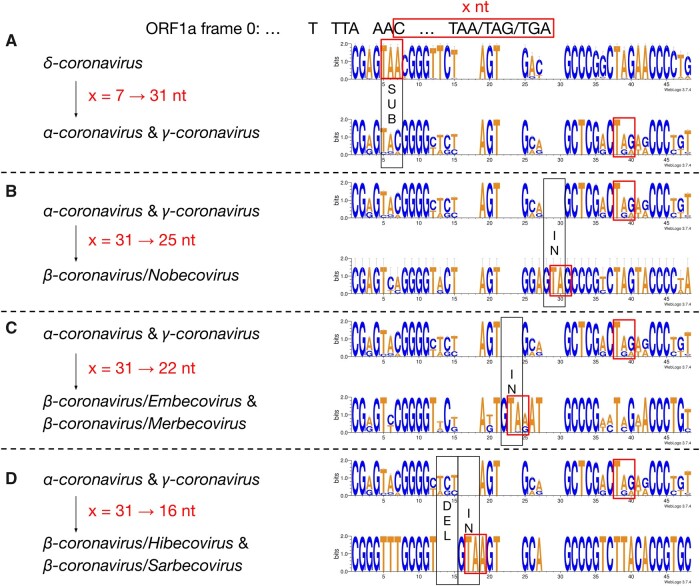
Nucleotide alignment of the overlap in coronaviruses with 7, 31, 25, 22, and 16 nt. The footprints of substitutions, insertions, and deletions are shown in black boxes, and labeled as “SUB,” “IN,” and “DEL”, respectively. The stop codon of ORF1a in each of the 7, 31, 25, 22, and 16 nt overlap coronaviruses is shown in a red box.

The abundance of SARS-CoV-2 sequencing data allows examining the substitution dynamics in population- and individual-level sequencing data. For population-level analysis, we identified variants in the PFE region from >1,550,000 genome sequences available from GISAID (see Materials and Methods section). However, because GISAID contains only assembled genomes, these data do not provide information about individual-level (intrasample) variation. Hence, we performed an additional detailed analysis of >55,000 samples generated with the COG-UK (Lythgoe et al. 2021) consortium (see Maier et al. (2021) for analysis details). A summary of results from both analyses is shown in table 1. There is little variation in the PFE region as the fraction of samples containing individual substitutions appears to be small (the two “Count” columns in table 1). Furthermore, the 30 out of 36 substitutions in table 1 are consistent with being a result of RNA editing events from APOBEC (Chen and MacCarthy 2017) or ADAR (Bazak et al. 2014) enzymatic complexes. The remaining six substitutions (all transitions) are predominantly located in the loop regions of the predicted PFE secondary structure (Huston et al. 2021) and thus likely have minimal effect on the secondary structure.

**Table 1.** Allelic Variants within the PFE Region are Called from Complete GISAID Genomes (population) and COG-UK (individual) Data.

Site	H	B	Reference	Population		Individual			
				Alternate	**Count** ^c^	Alternate	Min AF	Max AF	**Count** ^d^
13,425^a^			C	T	1,812	—	—	—	—
13,429^a^			C	T	460	—	—	—	—
13,430^a^			C	T	169	—	—	—	—
13,431^a^			C	T	517	—	—	—	—
13,432^b^			A	G	110	—	—	—	—
13,434^a^			G	A	213	—	—	—	—
13,43^a^			C	T/A	1,328/120	T	0.116	0.971	14
13,437^b^			T	C	195	C	0.985	0.988	5
13,440	S	S	G	A	116	—	—	—	—
13,443^b^↓			A	G/T	134/22	—	—	—	—
13,445^a^			C	T	680	T	0.068	0.970	25
13,447^a^			G	A	16	—	—	—	—
13,451^a^			C	T	393	T	0.941	0.977	19
13,457^a^			C	T	3,663	T	0.052	0.963	19
13,458^a^			G	—	—	A	0.069	0.970	6
13,458	L	L	G	T	1,220	T	0.080	0.976	6
13,481^b^			A	G	9	—	—	—	—
13,486^a^			C	T	1,656	T	0.055	0.965	7
13,487^b^			A	G	151	G	0.901	0.949	12
13,497^b^			A	G	434	—	—	—	—
13,498^a^			C	T	189	—	—	—	—
13,500^a^			C	T	243	—	—	—	—
13,504		S	G	T	102	—	—	—	—
13,505^a^			C	T	314	T	0.887	0.917	5
13,511		S	A	T/G/C	121/58/11	—	—	—	—
13,512		S	G	T	114	—	—	—	—
13,513^a^			G	A	342	—	—	—	—
13,514^a^			C	T	495	T	0.065	0.889	6
13,516^a^↑			C	T	4,272	T	0.101	0.840	49
13,525	S	S	A	C	104	—	—	—	—
13,526^b^			T	C	117	—	—	—	—
13,532^b^↓			A	G	742	—	—	—	—
13,535^a^↓			C	T	11,942	T	0.215	0.841	23
13,541^b^			T	C	26	—	—	—	—
13,547^a^			C	T	675	T	0.067	0.898	8
13,550^a^			C	T	2,346	T	0.878	0.921	11

a Potential APOBEC-edited sites;

b Potential ADAR-edited sites.

Site numbering is in 0-based coordinates.

c Out of 1,525,442 complete genome.

d Out of 55,163 individual samples. Locations of substitutions in a stem (S) or a loop (L) are based on structures predicted by Huston et al. (H) and Bhatt et al. (B). ↓ and ↑ highlight sites showing signatures of negative and positive selection, respectively (see table 2).

Through a comparative analysis of GISAID sequences, we found that several codons with non-negligible levels of variation (table 2) were subject to purifying selection: *RdRp*: 1 (A13,443>C/G), *RdRp*: 31 (13,532 A>G/C), *RdRp*: 32 (13,535 C>T). This is consistent with a strong degree of functional constraint. Interestingly, this analysis also identified a single codon: *RdRp*: T26I (13,516 T>C), which has been subject to pervasive positive selection since early 2021. Most of the sequences with this substitution are in the B.1.1.7 and B.1.177.77 lineages (this is a consensus majority mutation in B.1.177.77 and B.1.614 lineages). *RdRp*: T26I is present at low frequencies in many viral lineages but is increasing in prevalence in recent months (0.5–1.0% global prevalence in recent samples). Functional significance, if any, for this substitution has not been reported.

**Table 2.** Sites with Selection Signatures Identified using a Fixed Effects Likelihood Method on Internal Branches using SARS-CoV-2 Phylogeny Built from GISAID Sequences (FEL; [Kosakovsky Pond and Frost 2005]) α: synonymous substitution rate (maximum likelihood estimate, MLE), β: non-synonymous substitution rate (MLE), ω:β/α.

Codon	Nucleotide	α	β	ω	LRT *P* value
1	13,443	0	0	4.286	0.002
31	13,352	7.040	0	0	0.015
26	13,516	0	4.722	∞	0.004
32	13,535	5.205	0	0	0.035

Here, α < β signifies positive selection, while α > β is indicative of negative selection.

Our results provide an alternative way to assess exceptional conservation of the PFE using publicly available sequence data highlighting the fact that the entire PFE region appears to be under strong purifying selection. These patterns are similar to observations obtained from deep mutational scanning where any alteration at the majority of PFE region sites have deleterious effects on the frameshift efficiency (e.g., Carmody et al. 2021).

## Materials and Methods

### Coronavirus Entries Retrieval and Filter

The 35,152 coronaviral entries in the NCBI taxonomy database were sorted by length, and only those longer than 14,945 nt were kept, leaving a total of 4,939 genomes. The slippery site and following overlap sequences were manually inspected, in case the slippery site was incorrectly annotated. We further filtered out those entries if they contained no annotation information, or had gapped sequences in the overlap. 4,904 coronavirus entries were selected using this approach (supplementary table S1, Supplementary Material online).

### Amino Acid Alignment and Nucleotide Alignment of the Overlap Region

For all *δ-coronavirus* entries in supplementary table S1, Supplementary Material online, the first 13 amino acids of *ORF1b* were taken to generate a consensus sequence using WebLogo (Crooks et al. 2004). The same was done to *α-coronavirus* and* γ-coronavirus*. Within *β-coronavirus*, for *Nobecovirus*, *Embecovirus*, and *Merbecovirus*, the first 14 amino acids were used to build the consensus; for *Hibecovirus* and* Sarbecovirus*, the first 13 amino acids were used. In terms of the nucleotide sequence alignments, for each genus/subgenus, the nucleotide sequences used to generate the amino acids mentioned above were taken to make the nucleotide consensus sequence using WebLogo.

### Processing of GISAID Data

Each genome was subjected to codon‐aware alignment with the NCBI reference genome (accession number NC_045512) and then subdivided into ten regions based on CDS features: *ORF1a* (including *nsp10*), *ORF1b* (starting with nsp12), *S*, *ORF3a*, *E*, *M*, *ORF6*, *ORF7a*, *ORF8*, *N*, and *ORF10*. For each region, we scanned and discarded sequences containing too many ambiguous nucleotides to remove data with possible sequencing errors. Thresholds were 0.5% for the S gene, 0.1% for ORF1a and ORF1b genes, and 1% for all other genes. We mapped individual sequences to the NCBI reference genome (NC_045512) using a codon‐aware extension to the Smith‐Waterman algorithm implemented in the BioExt package (Pond et al. 2005; Gianella et al. 2011) and translated mapped sequences to amino‐acids. Codon sequences were next mapped onto the amino‐acid alignment. Variants were called directly. Selection analyses were performed using the protocols used previously (Faria et al. 2021; Tegally et al. 2021) based on the FEL analysis (Kosakovsky Pond and Frost 2005) within the HyPhy package (Kosakovsky Pond et al. 2019).

## Supplementary Material

Supplementary data are available at *Molecular Biology and Evolution* online.

## Supplementary Material

msab265_Supplementary_Data
