# Supplementary material for: Stepwise Evolution and Exceptional Conservation of ORF1a/b Overlap in Coronaviruses
Source: Mol Biol Evol. 2021 Sep 10;38(12):5678–84. doi: 10.1093/molbev/msab265 (PMC8499926; doi:10.1093/molbev/msab265)
Supplement: msab265_Supplementary_Data [file msab265_supplementary_data.zip › figS1.pdf]

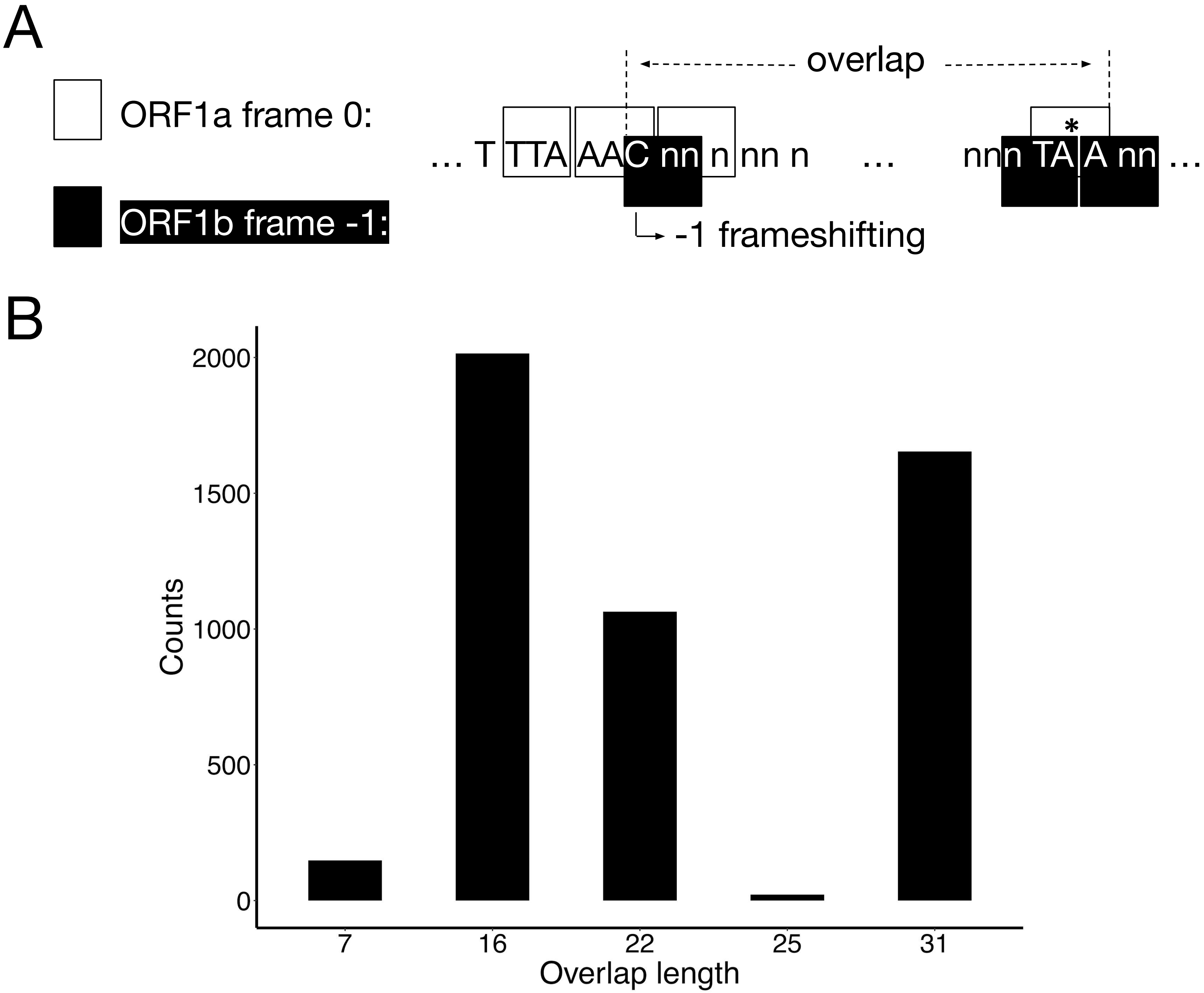

δ-coronavirus	7 nt	148	entries
β-coronavirus/Hibecovirus & *Sarbecovirus*	16 nt	2 & 2000	entries
β-coronavirus/Embecovirus & *Merbecovirus*	22 nt	420 & 613	entries
β-coronavirus/Nobecovirus	25 nt	14	entries
α-coronavirus & *γ-coronavirus*	31 nt	1210 & 426	entries
Unclassified	16 & 22 & 25 & 31 nt	71	entries
